# Phosphatidylserine-dependent structure of synaptogyrin remodels the synaptic vesicle membrane

**DOI:** 10.1038/s41594-023-01004-9

**Published:** 2023-05-22

**Authors:** Taekyung Yu, David Flores-Solis, Gunnar N. Eastep, Stefan Becker, Markus Zweckstetter

**Affiliations:** 1 German Center for Neurodegenerative Diseases (DZNE), Göttingen, Germany; 2 Department for NMR-based Structural Biology, Max Planck Institute for Multidisciplinary Sciences, Göttingen, Germany

**Keywords:** Solution-state NMR, Membrane proteins

## Abstract

Synaptic vesicles are small membrane-enclosed organelles that store neurotransmitters at presynaptic terminals. The uniform morphology of synaptic vesicles is important for brain function, because it enables the storage of well-defined amounts of neurotransmitters and thus reliable synaptic transmission. Here, we show that the synaptic vesicle membrane protein synaptogyrin cooperates with the lipid phosphatidylserine to remodel the synaptic vesicle membrane. Using NMR spectroscopy, we determine the high-resolution structure of synaptogyrin and identify specific binding sites for phosphatidylserine. We further show that phosphatidylserine binding changes the transmembrane structure of synaptogyrin and is critical for membrane bending and the formation of small vesicles. Cooperative binding of phosphatidylserine to both a cytoplasmic and intravesicular lysine-arginine cluster in synaptogyrin is required for the formation of small vesicles. Together with other synaptic vesicle proteins, synaptogyrin thus can sculpt the membrane of synaptic vesicles.

## Abstract (web summary)

The structure of synapses is critical for brain function. Yu et al. show how the synaptic vesicle protein synaptogyrin binds lipids and helps to ensure that vesicles are uniformly sized, allowing for precise storage of neurotransmitters.

## Main

Synaptic vesicles (SVs) are storage organelles of neurotransmitters located in the presynaptic bouton^1,2^. When SVs fuse with the presynaptic membrane, neurotransmitters are released to propagate chemical signaling between neurons^1–3^. To sustain rounds of synaptic transmission, SVs have to be recycled, requiring tight control of their biogenesis^4,5^. A characteristic property of SVs is their small uniform size, which is important for brain function, because it enables the storage of well-defined amounts of neurotransmitters and thus reliable synaptic transmission. The membrane of SVs is composed of a small number of lipids and organelle-specific, abundant transmembrane (TM) proteins^6^. Cell biological and biochemical studies have shown that both the SV TM proteins and lipids influence the morphology of SVs^1,7–11^. However, little is known how SV membrane proteins and lipids cooperate to sculpt SVs.

A major family of SV membrane proteins comprises the tetraspan vesicle membrane proteins synaptophysin and synaptogyrin^6,11^. Tetraspan vesicle membrane proteins are integral membrane proteins composed of four TM helices^11^. Multiple studies have linked synaptogyrin and synaptophysin to high membrane curvature and the formation of SVs^11–17^. Ectopic expression of synaptophysin in non-neuronal cells leads to the formation of small cytoplasmic vesicles^12^, while removal of synaptogyrin from SVs increases SV size^13^. Supporting this connection, the name synaptogyrin (from Greek γυρος, meaning circle) references the uniform spherical shape of SVs^14–16^. The synaptogyrin family consists of three isoforms, with synaptogyrin 1 and synaptogyrin 3 being neuronal^6,11^. Single and double knockout mice lacking synaptogyrin 1 and/or synaptophysin 1 did not have detectable morphological or biochemical changes in their SVs, but had reduced short- and long-term synaptic plasticity^16^. Mice in which synaptogyrin 1 and 3, as well as synaptophysin 1 and 2, were knocked out had elevated SV release probability^18^.

Synaptogyrins have been linked to several human diseases. Synaptogyrin 1 levels are decreased in the outer layer of the dentate gyrus in people with Alzheimer’s disease^19^. Synaptogyrin 3 plays an important role in presynaptic dysfunction induced by the protein Tau^20^, the protein that aggregates into the neurofibrillary tangles inside neurons, a pathological hallmark in people with Alzheimer’s disease^21^. Lowering synaptogyrin 3 expression rescued Tau-induced memory deficits in mice^22^. Mutations in synaptogyrin 1 have been identified in people with schizophrenia^23,24^. In addition, synaptogyrins have been associated with stroke and viral diseases^25,26^. Despite the important role of synaptogyrins in SV function and human disease, the structure of synaptogyrins, or any other tetraspan vesicle membrane protein, has remained enigmatic.

To gain insights into the molecular mechanisms that determine how SVs achieve their uniform morphology, we determined the high-resolution structure of synaptogyrin 1, studied its interaction with the SV-abundant lipid phosphatidylserine (PS), and investigated the ability of synaptogyrin to remodel the SV membrane. Our results establish a molecular mechanism for the sculpting of the SV membrane by synaptogyrin.

## Results

### Human synaptogyrin is a stable monomer

**Fig. 1:**
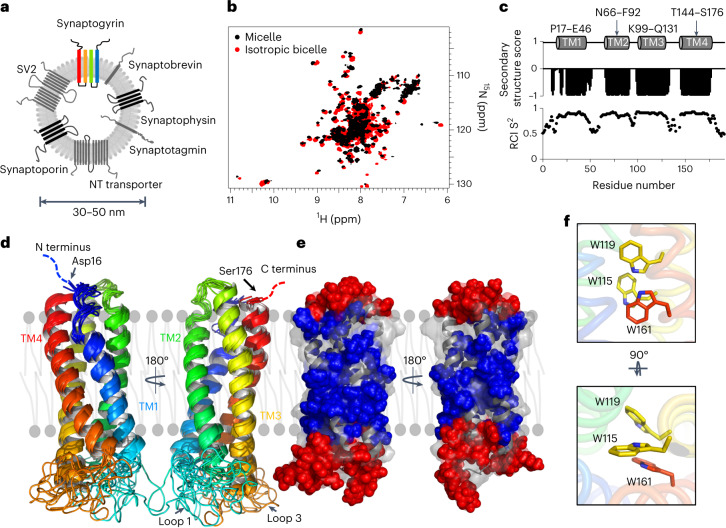
Structure of human synaptogyrin. **a**, Schematic representation of a SV (diameter: ~30–50 nm) with major TM proteins (NT transporter represents neurotransmitter transporters)^6^. Synaptogyrin is highlighted; other tetraspan vesicle membrane proteins are displayed in black. **b**, Superposition of ^1^H-^15^N TROSY-HSQC spectra of synaptogyrin in isotropic bicelles (red; DMPC/DHPC, *q* = 0.3) and n-undecyl β-maltoside (UDM) micelles (black). **c**, Residue-specific secondary structure score and random coil index (RCI S^2^) derived from the NMR chemical shifts of synaptogyrin. Four TM helices are identified: TM1 (P17–E46), TM2 (N66–F92), TM3 (K99–Q131) and TM4 (T144–S176). **d**, Ten lowest-energy structures of synaptogyrin in cartoon representation. The N- and C-terminal tails are located in the cytoplasm. **e**, Paramagnetic relaxation enhancement by 16-DSA and gadodiamide on synaptogyrin structure in UDM micelles. Residues with 16-DSA relaxation enhancement rates of >77 mM^−1^ s^−1^ (blue) are located inside the hydrophobic environment. Loop and tail residues have gadodiamide relaxation enhancement rates of >6.5 mM^−1^ s^−1^ (red). **f**, Interactions between conserved tryptophan residues inside the TM structure of synaptogyrin. Source data

To gain insight into the molecular basis of SV sculpting, we combined high-resolution structural analysis of synaptogyrin with membrane remodeling assays, nuclear magnetic resonance (NMR)-based lipid-binding experiments, and site-directed mutagenesis. We successfully overexpressed isoform b of human synaptogyrin 1 (hereafter named synaptogyrin; Fig. 1a) in *Escherichia coli* and directly extracted it from the membrane without refolding^27^. To enable high-resolution structural analysis by NMR spectroscopy, synaptogyrin was labeled with ^15^N and ^13^C and perdeuterated, and reconstituted into different detergents, as well as into isotropic bicelles formed by 1,2-dimyristoyl-*sn*-glycero-3-phosphocholine (DMPC) and 1,2-diheptanoyl-*sn*-glycero-3-phospocholine (DHPC)^28^. Bicelles are an excellent medium for studying membrane proteins^29–31^.

**Extended Data Fig. 1:**
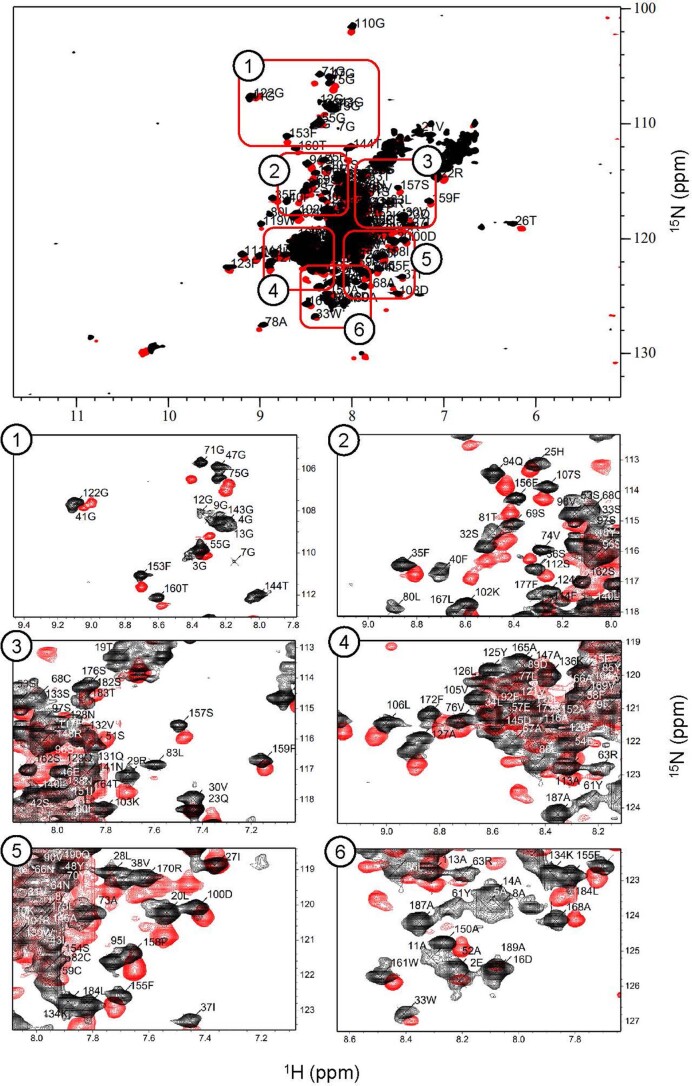
NMR spectroscopy of synaptogyrin. Superposition of ^1^H-^15^N TROSY-HSQC spectra of synaptogyrin reconstituted into undecylmaltoside (UDM; red), and isotropic bicelles (red; DMPC/DHPC, q = 0.3). Selected resonance assignments of synaptogyrin are indicated.

**Extended Data Fig. 2:**
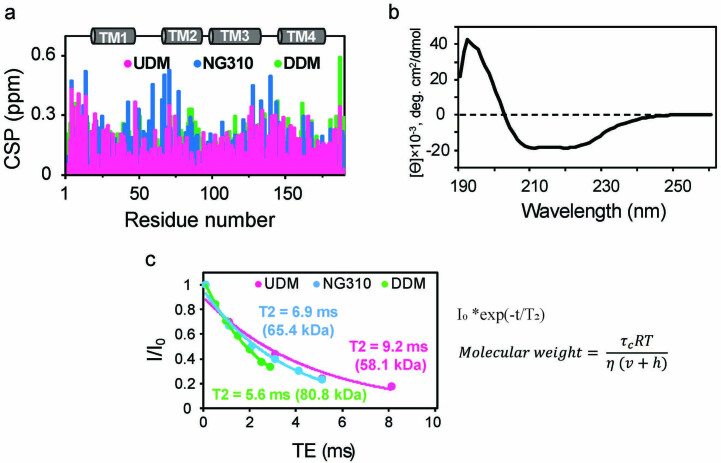
Biophysical characterization of synaptogyrin. **a**, Normalized averaged ^1^H-^15^N chemical shift differences (CSP) of synaptogyrin in isotropic bicelles (DMPC/DHPC, q = 0.3) when compared to synaptogyrin in the three different detergents. **b**, Circular dichroism (CD) spectrum of synaptogyrin in UDM micelles. **c**, Quantification of the transverse relaxation time *T*_*2*_ of the amide protons of synaptogyrin in the three different detergent micelles (UDM/pink, NG310/blue, DDM/green). *T*_*2*_ values derived from a single exponential fit to the decaying NMR signal intensities are indicated, together with the approximate molecular weight of the synaptogyrin/detergent assembly. Synaptogyrin 1b contains 191 residues (uniprot O43759-2) corresponding to a molecular weight of 21 kDa. The estimated molecular weights of the micelles without protein are 40~50 kDa for UDM, 65–70 kDa for DDM, and ~90 kDa for NG310 (please see Methods section). Source data

We observed high-resolution liquid-state NMR spectra with well-defined and dispersed synaptogyrin cross-peaks (Fig. 1b and Extended Data Fig. 1). Circular dichroism revealed the presence of α-helical structure (Extended Data Fig. 2), in agreement with four predicted TM helices^11^. We then estimated the average transverse NMR relaxation rates of the amide resonances of synaptogyrin, which are in agreement with a synaptogyrin monomer (Extended Data Fig. 2c). Synaptogyrin directly extracted from SVs by the detergent 3-[(3-cholamidopropyl)dimethylammonio]-1-propanesulfonate (CHAPS) is also monomeric^14^. The data demonstrate that synaptogyrin is a stable monomer in the investigated environments.

### High-resolution structure of synaptogyrin

**Extended Data Fig. 3:**
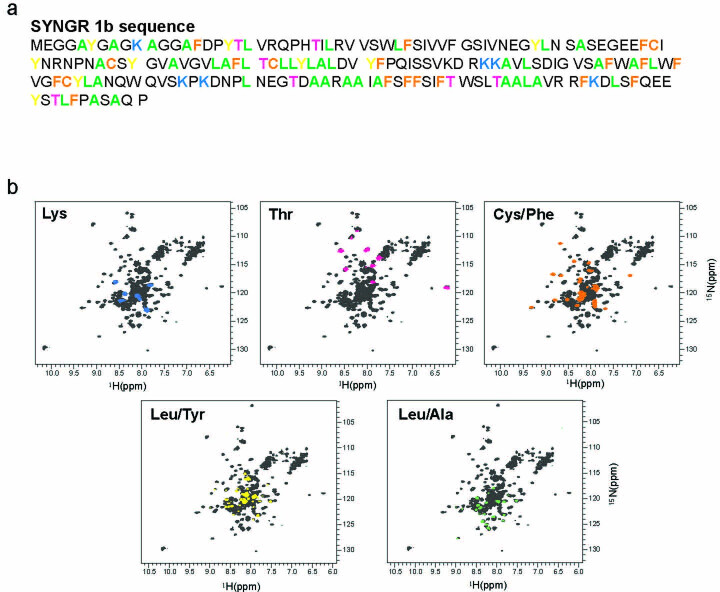
Selective labeling of synaptogyrin. **a**, Amino acid sequence of synaptogyrin 1b (SYNGR 1b) with color coding according to the selective isotope labeling of specific amino acids: lysine (blue), threonine (pink), cysteine/phenylalanine (orange), leucine/tyrosine (yellow), and leucine/alanine (green). **b**, Superpositions of the ^1^H-^15^N TROSY-HSQC spectra of amino acid type selective ^15^N-labeled synaptogyrin with the corresponding spectrum of the uniformly ^15^N-labeled protein. Spectra were recorded at 40 °C for synaptogyrin in UDM micelles on Bruker 800 (Cys/Phe), 900 (Leu/Tyr, Leu/Ala) and 950 MHz (Lys, Thr) NMR spectrometers.

**Extended Data Fig. 4:**
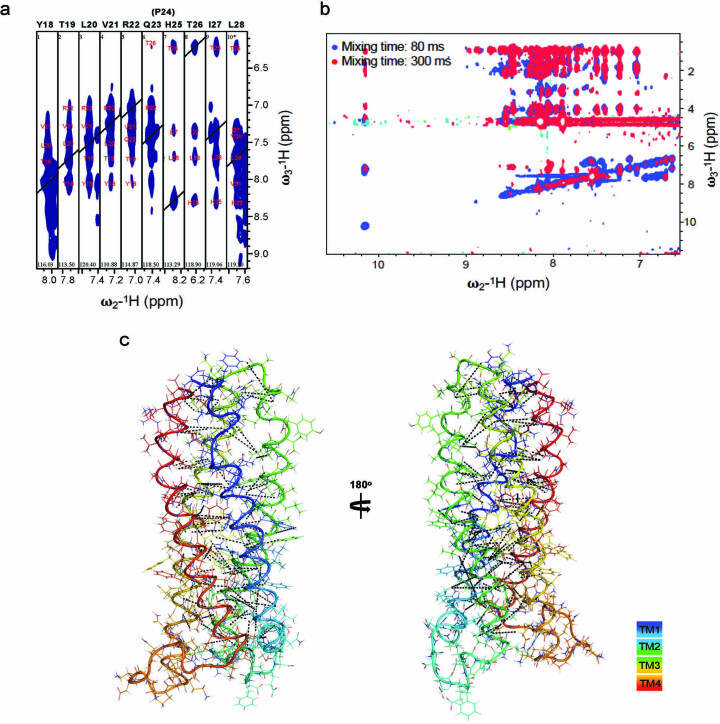
Backbone assignment and NOE restraints. **a**, Strips from a 3D ^15^N-edited NOESY-HSQC (NOE mixing time of 200 ms; U-[^2^H, ^15^N]-labeled synaptogyrin) highlighting the assignment of a part of TM1. Cross-peaks establishing inter-residual connectivity are labeled. **b**, Superposition of 2D ^1^H-^1^H NOESY planes extracted from 3D ^15^N-edited NOESY-HSQC spectra (NOE mixing times of 80 ms (blue) and 300 ms (red)) recorded on synaptogyrin in n-dodecyl-d25-β-D-maltopyranoside (d25-DDM, that is deuterated DDM) micelles. **c**, Long-range NOEs (black dashed lines) mapped onto the 3D structure of synaptogyrin.

To resolve the three-dimensional (3D) structure of synaptogyrin to high resolution, we determined the protein’s backbone resonance assignment. NMR assignment of multispan α-helical TM proteins is complicated by strong signal overlap, which arises from the large line widths and limited chemical shift dispersion in α-helical TM regions^32^. We overcame this challenge by combining multi-dimensional NMR experiments optimized for high-molecular-weight membrane proteins with amino acid type-specific labeling (Supplementary Table 1 and Extended Data Figs. 3 and 4a)^32,33^. Through this combined approach, we achieved backbone assignment for 98% of the residues. On the basis of the assigned NMR chemical shifts, we identified four TM helices formed by residues P17–E46 (TM1), N66–F92 (TM2), K99–Q131 (TM3) and T144–S176 (TM4) (Fig. 1c). The three loops between the TM helices, as well as the amino- and carboxy-terminal tails, are disordered (Fig. 1c). Comparison of the ^1^H and ^15^N chemical shifts of synaptogyrin in isotropic bicelles and detergent micelles showed that chemical shift changes occur predominantly in the disordered regions (Extended Data Fig. 2a).

**Extended Data Fig. 5:**
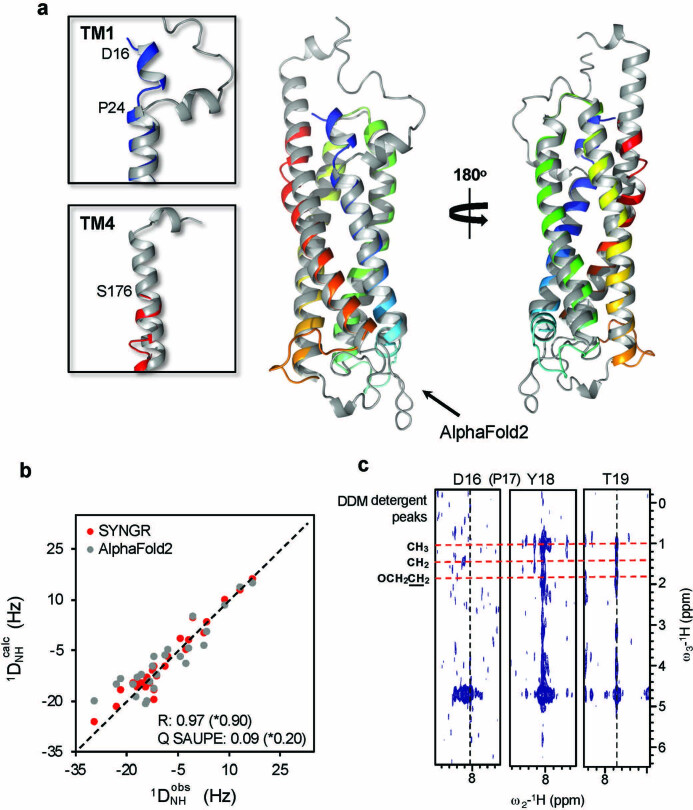
Comparison of the experimental NMR-based structure of synaptogyrin 1b with the structure predicted by AlphaFold2. **a**, Alignment of the lowest-energy NMR structure (color coding as in Fig. 1d) with the structure predicted using the Google collaborative notebook for AlphaFold2 prediction (https://colab.research.google.com/drive/1LVPSOf4L502F21RWBmYJJYYLDlOU2NTL) (grey). The two insets highlight the regions that differ most strongly between the two structures. In the experimentally determined structure, TM4 ends at S176 (lower inset), in agreement with the experimental NMR chemical shifts (Fig. 1c). In contrast, TM4 extends to L184 and is followed by another short helix comprising residues 187–189 in the AlphaFold2-predicted structure. Notably, a TM4 helix up to L184 would extend significantly into the cytosol. The second major difference (upper inset) relates to the orientation of the short helical region at the N-terminus of TM1, which precedes the kink at P24. In the NMR-derived structure, this TM1-part contacts TM4, while in the AlphaFold2-predicted structure it makes a ~90° turn. The ~90° turn would place residue D16 inside the lipid environment. In n-dodecyl-ß-D-maltoside (DDM) micelles, commonly used for solubilization of membrane proteins, such a positioning is unlikely because D16 does not show NOE contacts with the aliphatic protons of DDM in contrast to Y18 and T19 (please see panel (c)). **b**, Correlation between experimental ^1^H-^15^N RDCs and values back-calculated using singular value decomposition from the experimentally-determined 3D structure of synaptogyrin 1b (red) or from the AlphaFold2-predicted structure (grey). The analysis is based on 28 RDCs from residues located in the four TM helices. Pearson correlation values *R* (RDC quality factor *Q*) between experimental and back-calculated values are 0.97 (*Q* = 0.09; Da(NH) = 21.2 Hz; Rhombicity = 0.25) for the experimentally determined structure, and 0.90 (*Q* = 0.20; Da(NH) = 17.2 Hz; Rhombicity = 0.15) for the AlphaFold2-predicted structure. **c**, Strips from the 3D ^15^N-edited NOESY-HSQC showing intermolecular NOEs between the methyl and methylene groups of UDM and the synaptogyrin residues Y18 and T19, but not D16. Source data

**Extended Data Fig. 6:**
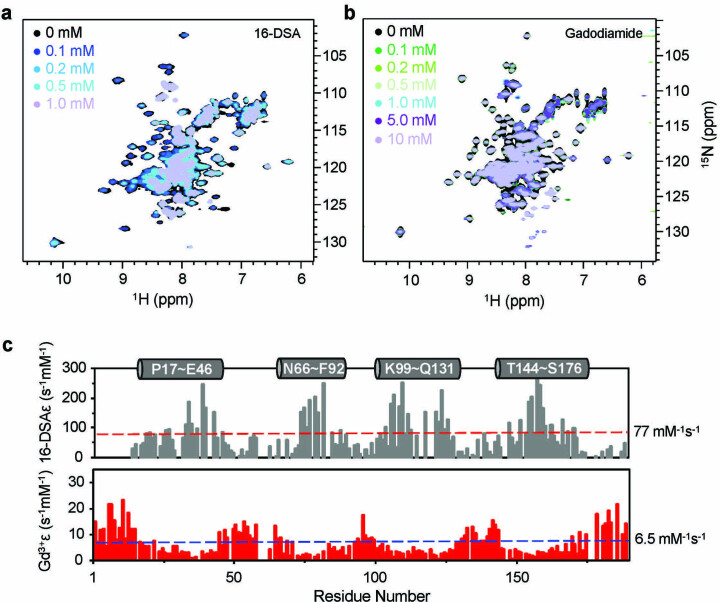
Solvent accessibility probed by paramagnetic relaxation enhancement. **a, b**, Superposition of ^1^H-^15^N TROSY-HSQC spectra of ^15^N/^2^H-labeled synaptogyrin in UDM micelles titrated with increasing concentrations of 16-DSA (a) or gadodiamide (b) up to 1.0 mM and 10 mM, respectively. **c**, Residue-specific relaxation enhancement values obtained from the titration with 16-DSA (grey, top) and gadodiamide (red, bottom). Residues with 16-DSA relaxation enhancements > 77 mM^−1^s^−1^ (dashed red line) are located in the hydrophobic interior of the micelle. Residues with gadodiamide relaxation enhancements > 6.5 mM^−1^s^−1^ (dashed blue line) are solvent exposed. The location of the four TM helices is indicated above. Source data

We then recorded side chain-specific NMR experiments and assigned 83% of the side chain protons. Distance restraints were subsequently derived from 3D NOESY spectra (Supplementary Table 2 and Extended Data Fig. 4b,c) and paramagnetic relaxation enhancement data of a single-cysteine mutant protein tagged with the nitroxide spin label MTSL (S-(1-oxyl-2,2,5,5-tetramethyl-2,5-dihydro-^1^H-pyrrol-3-yl)methyl methanethiosulfonate). In addition, we measured ^1^H-^15^N residual dipolar couplings (RDCs) of synaptogyrin weakly aligned in a polyacrylamide gel. The combined NMR data were subjected to structure calculation in Rosetta^34^. The ten resulting lowest-energy structures have a r.m.s. deviation (r.m.s.d.) of 0.5 Å (Fig. 1d, Table 1 and Extended Data Fig. 5). Paramagnetic relaxation confirmed the hydrophobic burial of the TM region and the solvent accessibility of the loops and tails (Fig. 1e and Extended Data Fig. 6).

**Table 1:** NMR constraints and structural statistics for the ensemble of ten best energy structures of synaptogyrin

Protein	
**NMR distance and dihedral constraints**	
Distance constraints	
Total NOE	474
Sequential (|*i* – *j*| = 1)	259
Medium-range (|*i* – *j*| < 4)	130
Long-range (|*i* – *j*| > 5)	85
Total dihedral angle restraint	
*ϕ* + *ψ* dihedral angle restraints	144
Residual dipolar coupling (RDC) restraint	
Total RDCs	28
*Q*	0.2
Paramagnetic relaxation enhancement (PRE)	
Total PREs	68
**Ramchandran plot regions**	
Residues in most favored regions (%)	98.6
Residues in additionally allowed regions (%)	1.4
Residues in generously allowed regions (%)	0.0
Residues in disallowed regions (%)	0.0
**Average pairwise r.m.s.d. (Å)**	
Backbone	0.49 ± 0.18 Å

^a^The r.m.s.d. was calculated among the ten structures for residues 16–176.

**Extended Data Fig. 7:**
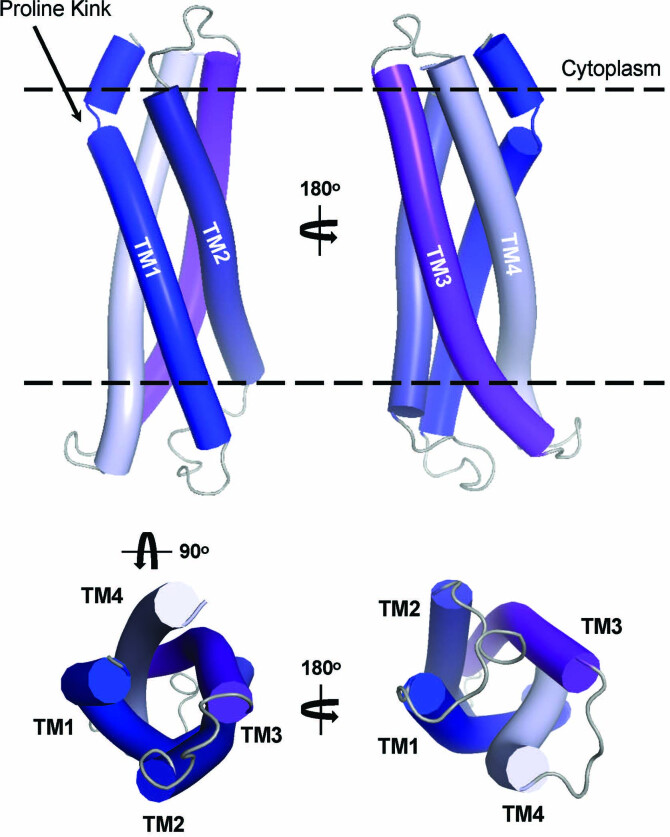
Synaptogyrin structure. Side- and top/bottom-views of the lowest-energy structure of synaptogyrin in which the TM helices are represented by tubes. Black dotted lines indicate approximate membrane boundaries.

The three-dimensional structure reveals a tight bundle of four α-helices (Fig. 1d). Previous studies have shown that the N- and C-terminal tails are located on the cytoplasmic side, and the TM1–TM2 and TM3–TM4 loops point to the interior of SVs^11^. The four TM helices are curved and twist around a central axis (Fig. 1d and Extended Data Fig. 7). Helix TM1 kinks at proline P24. The short loop between TM2 and TM3 is well defined and is the least flexible loop, according to the chemical-shift-derived flexibility parameter (Fig. 1c).

**Fig. 2:**
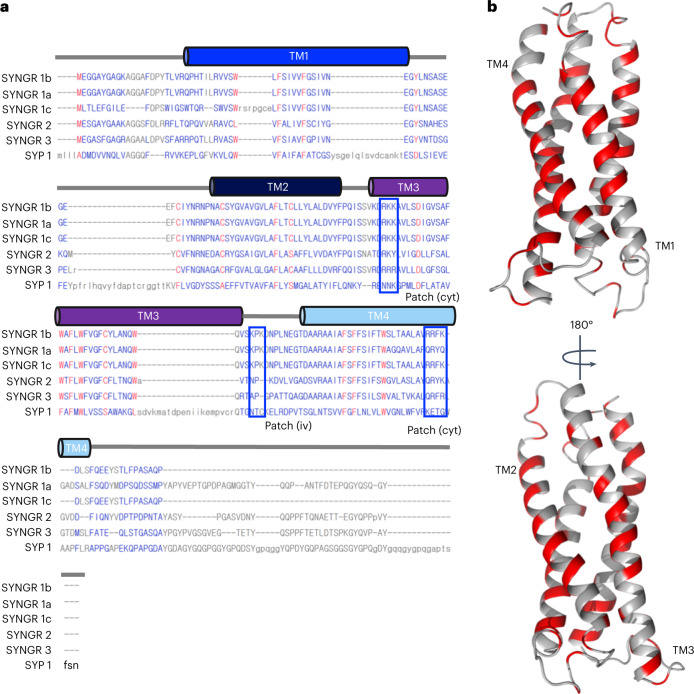
Sequence conservation of synaptogyrins. **a**, Sequence alignment of the different isoforms of synaptogyrin together with the amino acid sequence of synaptophysin (SYP 1). The lysine-arginine patches on the cytoplasmic (cyt) and the intravesicular (iv) membrane side are highlighted by blue boxes. **b**, Mapping of conserved residues (red) onto the 3D structure of synaptogyrin.

The sequence compositions of the four TM helices of different synaptogyrin isoforms, as well as of the tetraspan vesicle membrane protein synaptophysin, are well conserved (Fig. 2a,b). Synaptogyrin 1a–c and synaptogyrin 3 contain four cysteine residues, with C59 being located in the TM1–TM2 loop, C68 and C82 in TM2, and C124 in TM3. In synaptogyrin 2, C82 in TM2 is replaced by serine (Fig. 2a). The 3D structure of synaptogyrin 1b shows that the cysteines are spatially separated and do not form disulfide bonds. The 3D structure of synaptogyrin 1b further reveals contacts between the aromatic rings of W115 and W119 from TM3 together with W161 from TM4 (Fig. 1f). These three tryptophan residues are fully conserved in different synaptogyrin isoforms (Fig. 2a).

### Synaptogyrin sculpts small vesicles

**Fig. 3:**
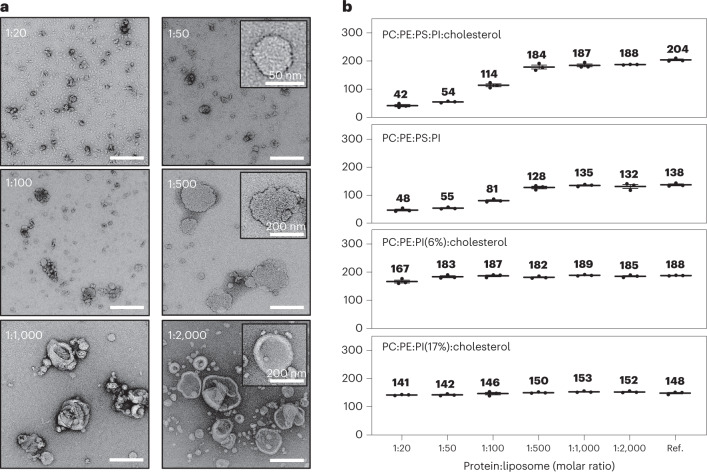
Synaptogyrin sculpts SV-sized vesicles. **a**, Negative-stain electron micrographs (scale bar, 200 nm) of liposomes and vesicles comprising phosphatidylcholine (PC), phosphatidylethanolamine (PE), phosphatidylserine (PS), phosphatidylinositol (PI) and cholesterol (PC:PE:PS:PI:cholesterol, 40:32:12:5:10)^35^ with increasing concentrations of synaptogyrin. Insets display individual liposomes or vesicles. **b**, Average DLS-derived diameter of liposomes and vesicles without or with increasing concentrations of synaptogyrin. Lipid compositions (wt/wt): top panel, PC:PE:PS:PI:cholesterol, 40:32:12:5:10; second panel, PC:PE:PS:PI, 43:34:15:7; third panel, PC:PE:PI:cholesterol, 47:36:6:10; bottom panel, PC:PE:PI:cholesterol, 40:32:17:10. Error bars represent s.d., based on three repeated measurements. Source data

**Extended Data Fig. 8:**
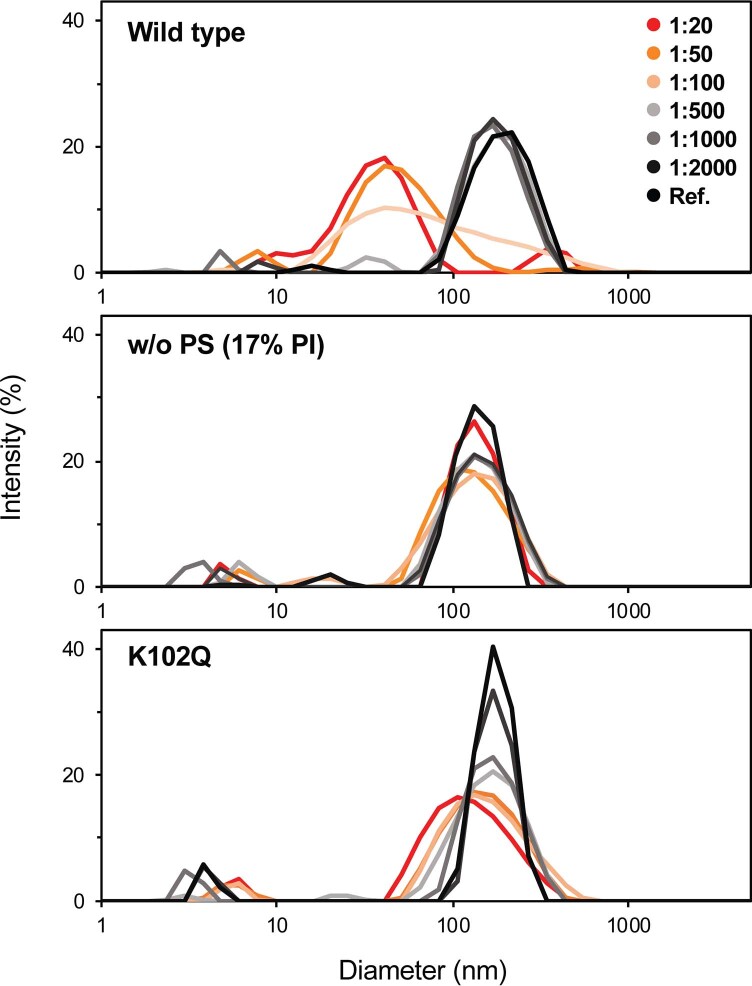
DLS histograms of liposomes. DLS histograms of liposomes without and with increasing concentrations of wild-type synaptogyrin (top and middle panel) or K102Q mutant synaptogyrin (bottom panel). The color code for different protein:lipid molar ratios is indicated in the top panel. In the top and bottom panel, the lipid composition (wt/wt) is PC:PE:PS:PI:cholesterol = 40:32:12:5:10. In the middle panel, the lipid composition is PC:PE:PI:cholesterol = 40:32:17:10.

**Extended Data Fig. 9:**
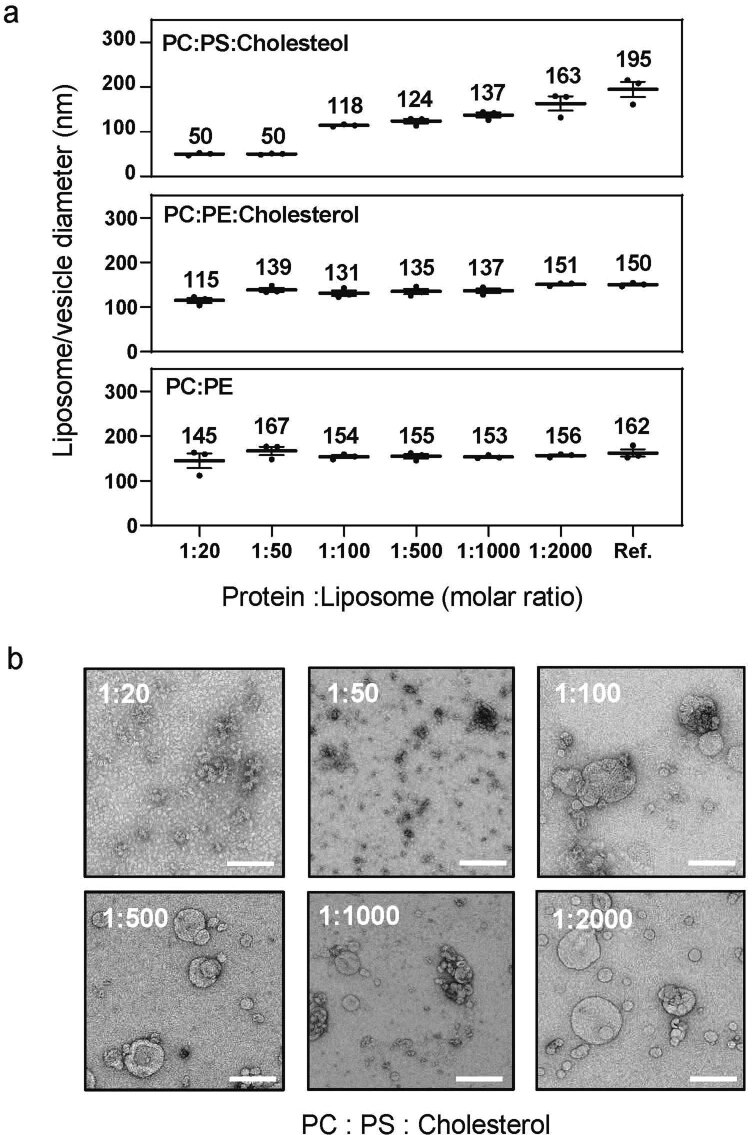
Influence of lipid composition on the ability of synaptogyrin to remodel liposomes. **a**, Average DLS-derived diameter of liposomes/vesicles without or with increasing concentrations of synaptogyrin. Lipid compositions (wt/wt): PC:PS:Cholesterol = 73:17:10 (upper panel)^2^, PC:PE:Cholesterol = 50:40:10 (middle panel), and PC:PE = 55:45 (bottom panel). Error bars represent std based on three repeated measurements. **b**, Negative-stain electron micrographs (of liposomes/vesicles composed of 73 % PC, 17 % PS and 10 % cholesterol (wt/wt) containing increasing concentrations of synaptogyrin (protein:lipid molar ratios are indicated). Scale bar, 200 nm. Source data

Next, we prepared liposomes that mimic the lipid composition of SVs (SVs have a diameter of ~30–50 nm)^6,35,36^. Liposomes of different sizes were visible in electron micrographs (Fig. 3a and Extended Data Figs. 8 and 9). Using dynamic light scattering (DLS), we estimated that the average diameter of the liposomes was ~200 nm (Fig. 3b and Extended Data Figs. 8 and 9). We then reconstituted synaptogyrin at increasing concentrations into the liposomes. For protein:lipid molar ratios of 1:2,000, 1:1,000 and 1:500, the average liposome diameter only slightly decreased (Fig. 3b). At the protein:lipid ratio of 1:500, however, the surface of the liposomes appeared more rugged when compared with that at lower synaptogyrin concentrations (Fig. 3a, middle row). When we further increased the concentration of synaptogyrin to protein:lipid molar ratios of 1:50 and 1:20, we observed only small vesicles, with average DLS-derived diameters of ~54 nm and ~42 nm, respectively (Fig. 3b, top row, and Extended Data Fig. 8). Synaptogyrin thus dramatically remodels SV-like membranes, generating small vesicles with a diameter comparable to SVs.

The SV membrane is rich in cholesterol^6,36^, and cholesterol binds to the tetraspan SV protein synaptophysin^8^. To determine whether cholesterol is required for synaptogyrin’s membrane-sculpting activity, we repeated the experiments using liposomes without cholesterol. Without synaptogyrin and cholesterol, the DLS-derived liposome diameter was ~130–140 nm (Fig. 3b), smaller than in presence of cholesterol, which is known to increase membrane stiffness and thus makes membranes harder to bend^37^. When we reconstituted synaptogyrin at increasing concentrations, the DLS-derived diameter decreased, and ~40- to 50-nm-large vesicles formed (Fig. 3b). Cholesterol is thus not required for synaptogyrin-induced sculpting of the SV-like membrane.

The most abundant negatively charged lipid in the SV membrane is PS^6,35^, a phospholipid with unique physical and biochemical properties^38^. To investigate the role of PS, we replaced it with the anionic lipid phosphatidylinositol (PI) and tested two different PI concentrations. Electron microscopy and DLS showed that, even with 17% PI and at the highest concentration of synaptogyrin, no membrane remodeling occurred (Fig. 3b, lower panel). Remodeling of the SV-like membrane is thus specific for both synaptogyrin and PS.

### Membrane remodeling depends on synaptogyrin-phosphatidylserine interaction

**Fig. 4:**
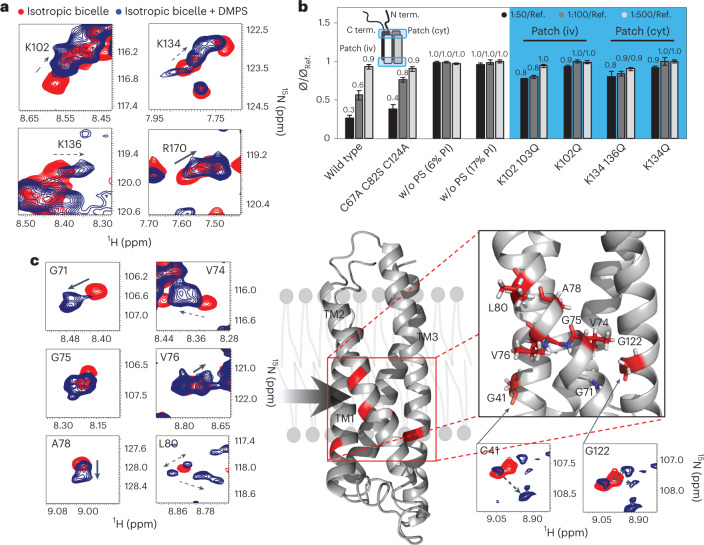
Membrane remodeling depends on synaptogyrin-phosphatidylserine interaction. **a**, Selected lysine and arginine cross-peaks in ^1^H-^15^N TROSY-HSQC spectra of synaptogyrin in isotropic bicelles (DMPC/DHPC, *q* = 0.3) without (red) or with DMPS (20%; blue). **b**, Change in average DLS-derived diameters (*Φ*) of liposomes and vesicles in the presence of increasing concentrations of wild-type synaptogyrin or different synaptogyrin mutants (protein:lipid molar ratios of 1:50 (dark gray), 1:100 (gray) and 1:500 (light gray)) when compared with liposomes without protein (*Φ*_Ref._). Lipid composition (wt/wt) when not noted otherwise: PC:PE:PS:PI:cholesterol, 40:32:12:5:10. Synaptogyrin displays a lysine-arginine patch at both the intravesicular (iv) and the cytoplasmic (cyt) membrane side (displayed as blue boxes) containing K102 and K103, and K134 and K136, respectively. The data for wild-type synaptogyrin in liposomes lacking PS are shown for comparison. The protein containing the substitutions C68A, C82S and C124A serves as control. Error bars represent s.d., based on three repeated measurements. **c**, DMPS binding induces different TM structural states in synaptogyrin, as evidenced by multiple cross-peaks for individual residues. Cross-peaks observed in isotropic bicelles without and with 20% DMPS are displayed in red and blue, respectively. Tentative cross-peak assignments in the presence of DMPS are indicated by dashed arrows. Residues that display multiple cross-peaks in the presence of DMPS cluster together in the synaptogyrin structure (marked in red). Source data

**Fig. 5:**
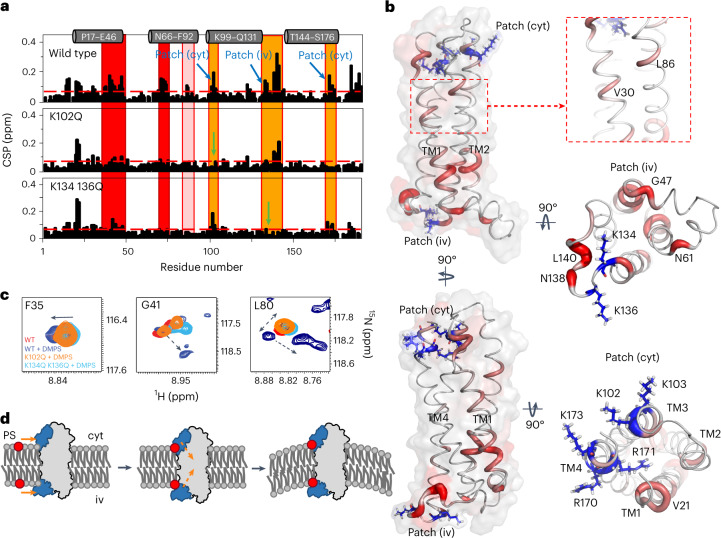
Phosphatidylserine shapes synaptogyrin TM structure. **a**, Residue-specific changes in the ^1^H-^15^N chemical shifts (CSP, chemical shift perturbation) of wild-type (top) and mutant synaptogyrin (K102Q, middle; K134Q K136Q, bottom) in isotropic bicelles (DMPC/DHPC, *q* = 0.3) without and with 20% DMPS. The dashed red line marks the average of DMPS-induced CSPs observed in wild-type synaptogyrin. Substitution sites are marked by green arrows. The location of the cytoplasmic (cyt) and the intravesicular (iv) lysine-arginine patch is marked in orange. Red boxes mark the location of residues that display both chemical shift changes and peak splitting. L86 and A87 in TM2 (pink box) also display DMPS-induced CSPs. The location of the four TM helices is shown above. **b**, CSPs induced by DMPS in wild-type synaptogyrin (**a**, top panel) mapped onto the 3D structure. Larger CSPs are represented by increased tube thickness and a brighter red color. Lysine and arginine residues located on the cytoplasmic (cyt) and the intravesicular (iv) membrane side are displayed with side chains (blue). **c**, Superpositions of cross-peaks from the ^1^H-^15^N TROSY-HSQC spectra of wild-type synaptogyrin without (red) or with DMPS (blue), as well as the two synaptogyrin mutants in the presence of DMPS (K102Q synaptogyrin, orange; K134Q K136Q synaptogyrin, cyan). Tentative cross-peak assignments in the presence of DMPS are indicated by the dashed arrows. **d**, Scheme for the remodeling of the SV membrane by synaptogyrin. Simultaneous binding of phosphatidylserine (PS; red) to the lysine-arginine cluster (blue) on the cytoplasmic and the intravesicular side of synaptogyrin (left) induces conformational changes in the TM structure of synaptogyrin (gray and orange, middle) that trigger the bending of the SV membrane (right). Source data

**Extended Data Fig. 10:**
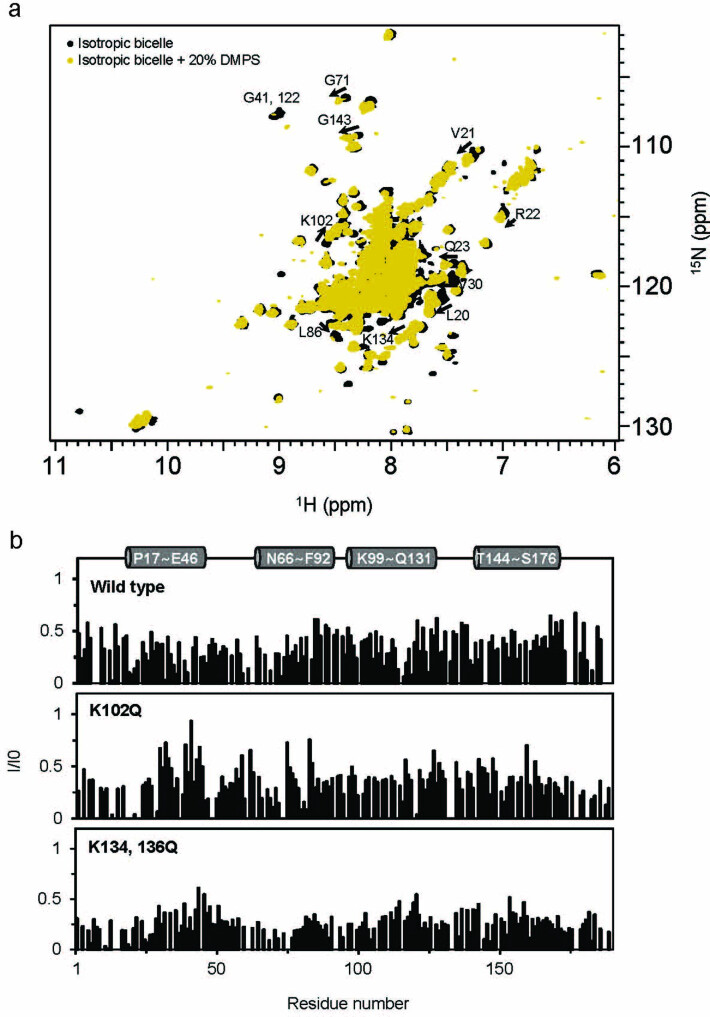
NMR spectroscopy of wild-type and mutant synaptogyrin. **a**, Superposition of ^1^H-^15^N TROSY-HSQC spectra of ^15^N/^2^H-labeled synaptogyrin reconstituted into isotropic bicelles (DMPC/DHPC, q = 0.3) without (black) or with 20 % DMPS (10-fold molar excess of DMPS over protein; gold). Strongly perturbed cross-peaks are labeled by their assignment. **b**, Residue-specific intensity ratios of ^1^H-^15^N cross-peaks of wild-type (top), or mutant synaptogyrin (K102Q, middle; K134Q/K136Q, bottom), in isotropic bicelles (DMPC/DHPC, q = 0.3) without (I_0_) and with 20 % DMPS (I). Source data

To gain molecular insights into synaptogyrin-based SV membrane sculpting, we reconstituted ^15^N-^2^H-labeled synaptogyrin into bicelles formed by DMPC and DHPC, as well as into DMPC/DMPS/DHPC-bicelles with 1,2-dimyristoyl-sn-glycero-3-phospho-L-serine (DMPS) in 40-fold molar excess over synaptogyrin. The ^1^H-^15^N correlation spectra of synaptogyrin in the two bicelle environments closely superimposed with selected cross-peaks displaying DMPS-induced changes (Extended Data Fig. 10 and Figs. 4a and 5a, top panel). DMPS changes both the chemical shifts and the intensities of several residues (Fig. 4a,c and Extended Data Fig. 10). In addition, the cross-peaks of some residues, such as G41, G71, G75, A78, L80 and K136, were split into 2–3 new signals in the presence of DMPS (Fig. 4a,c). K136 together with K134 forms a positively charged patch on the intravesicular side (Fig. 5b). K102 and R170, which are located on the cytoplasmic side of synaptogyrin (Fig. 5b), were also perturbed in the presence of DMPS (Fig. 4a).

Residue-specific analysis of the chemical shift changes confirmed that both lysine- and arginine-rich regions were perturbed in the presence of DMPS (Fig. 5a, top panel). The DMPS-induced chemical shift changes of residues 138–140 might be caused by binding of DMPS to the nearby K134 or K136 (Fig. 5b). The NMR signal perturbations support binding of DMPS, likely through its negatively charged head group, to the two positively charged lysine-arginine clusters located on the cytoplasmic and the intravesicular side of synaptogyrin.

We next probed the importance of PS binding to synaptogyrin by combining site-specific protein mutation with membrane-remodeling assays (Fig. 4b). Either one or two positively charged residues in the two lysine-arginine clusters were mutated to glutamine. None of the charge-attenuated synaptogyrin mutants was able to remodel liposomes into SV-sized vesicles (Fig. 4b, Extended Data Fig. 8 and Supplementary Fig. 1). By contrast, a control mutant protein, in which the three cysteines, C68, C82 and C124, were mutated, retained its membrane-sculpting activity (Fig. 4b). The experiments demonstrate that the two lysine-arginine clusters of synaptogyrin are required for the protein’s ability to remodel the SV-like membrane.

Besides the PS-associated changes of the NMR signals in the two lysine-arginine clusters, we found pronounced signal perturbations for residues 68–80 in TM2, as well as for residues 14–23 and 35–48 in TM1 (Fig. 5a, top panel, b). In addition, we observed two to four cross-peaks for individual residues in the regions 35–41 and 71–80 in the presence of PS (Fig. 4c). For example, a single cross-peak is present for L80 without DMPS that splits into four cross-peaks with PS. None of the 35–48 or 68–80 residues is positively charged, making a direct interaction of these residues with PS unlikely. Instead, the detection of multiple NMR signals for individual residues provides evidence for the formation of distinct TM structural states, which are in slow exchange on the NMR time scale. In the high-resolution structure of synaptogyrin, residues 35–48 and 68–80 cluster together at the cross-over of TM1 and TM2 (Figs. 4c and 5b). Binding of PS to the two lysine-arginine patches thus induces a conformational rearrangement of the TM1–TM2 interface of synaptogyrin.

### Phosphatidylserine binding reshapes synaptogyrin structure

To gain further insight into the connection between PS binding and conformational changes in synaptogyrin, we prepared the ^15^N-^2^H-labeled mutant proteins synaptogyrin-K102Q and synaptogyrin-K134Q K136Q. We reconstituted the mutant proteins into DMPC/DHPC-bicelles without or with DMPS and recorded two-dimensional ^1^H-^15^N correlation spectra. For both mutant proteins, a 40-fold excess of DMPS did not induce chemical shift changes in the respective mutated lysine-arginine cluster (Fig. 5a, middle and lower panels). In addition, residues 14–16 at the N terminus of TM1, which are close to the cytoplasmic K102, K103 and R170 patch, were not perturbed by DMPS in the synaptogyrin-K102Q (Fig. 5a, middle panel, b). For all three proteins, DMPS induced changes for residues 20–23, suggesting a direct interaction of DMPS with R22 (Fig. 5a and Extended Data Fig. 10b). Notably, a substitution in either the cytoplasmic or the intravesicular lysine-arginine cluster did not abolish the DMPS-induced perturbations in the cluster on the other side of the membrane (Fig. 5a). For example, DMPS caused chemical shift changes for residues 101–103 in synaptogyrin-K134Q K136Q (Fig. 5a, lower panel). DMPS thus binds independently to the two lysine-arginine clusters located on the cytoplasmic and the intravesicular side of synaptogyrin.

Alterations in the cytoplasmic or the intravesicular lysine-arginine cluster abolished the ability of synaptogyrin to remodel liposomes into small vesicles (Fig. 4b). Therefore, DMPS-induced changes in the NMR spectra that are inhibited by both mutations are particularly interesting. This is the case for the DMPS-induced chemical shift perturbation and peak splitting of residues 68–80 in TM2 and 35–48 in TM1. For both synaptogyrin-K102Q and synaptogyrin-K134Q K136Q, the presence of DMPS did not cause perturbations of residues 68–80, and smaller changes for residues 35–48 (Fig. 5a). DMPS binding to the non-mutated, solvent-accessible lysine-arginine cluster thus did not generate multiple structural states for the residues in the TM regions. Instead, the peak positions of these residues in the mutant proteins in the presence of DMPS were similar to those of the wild-type protein without DMPS (Fig. 5c and Supplementary Fig. 2). In addition, p.K102Q as well as p.K134Q and p.K136Q abolished or attenuated the DMPS-associated changes of residues ^85^YLA^87^ in TM2 and V30 in TM1 (Fig. 5a,b). When we inhibited binding of PS to either the cytoplasmic or the intravesicular lysine-arginine cluster, we blocked the phospholipid-induced changes at the TM2–TM1 interface (Fig. 5b), and the ability of synaptogyrin to remodel membranes into small vesicles (Fig. 4b).

## Discussion

Protein-lipid interactions govern the functional properties of cellular membranes and have been suggested to shape SVs^1,7–11,39,40^. An important morphological feature of SVs is their uniform size, which ensures that well-defined amounts of neurotransmitters are stored in each SV^1^. Although several complementary mechanisms may act in concert to enable a uniform morphology of SVs, our study establishes a connection between the 3D structure of the tetraspan vesicle membrane protein synaptogyrin, its interaction with the abundant SV-lipid PS and the formation of small vesicles. Binding of PS to the two lysine-arginine clusters located at the cytoplasmic and intravesicular side of synaptogyrin induces a rearrangement of the TM2–TM1 interface (Fig. 4c). Binding of PS to either the cytoplasmic or the intravesicular side is not sufficient to trigger the structural changes in synaptogyrin (Fig. 5a). Only when PS binds to both clusters, the TM structure of synaptogyrin is changed, and small vesicles are formed (Figs. 3 and 4b,c). This suggests that the binding processes of PS to the cytoplasmic and the intravesicular basic patch work together. The additive/cooperative action might be enabled through the direct interaction of the cytoplasmic lysine-arginine cluster with the N-terminal end of TM1 (Fig. 5b, cyt view), and of the intravesicular basic patch with the N-terminal end of TM2 (Fig. 5b, iv view).

While the PS-associated generation of multiple cross-peaks for residues at the TM1–TM2 interface indicates that PS binding changes the TM structure and likely results in two to three different synaptogyrin TM1–TM2 conformations, which are in slow exchange on the NMR chemical shift time scale (Fig. 4c), the high-resolution structures of these synaptogyrin states are currently unknown. Determination of these structures is complicated not only by the underlying dynamics, but also by the high molecular weight of the synaptogyrin-bicelle assembly that is a challenge for NMR studies. In addition, the strong aliphatic resonances of PS in NOE spectra complicates the extraction of NOE contacts for synaptogyrin in complex with PS. In the absence of a high-resolution structure of synaptogyrin with PS molecules simultaneously bound to both the cytoplasmic and the intravesicular basic patch, we can only hypothesize that PS binding changes the relative orientation of the TM1 and TM2 helices and thus induces a more curved synaptogyrin structure, which promotes the bending of the membrane (Fig. 5d).

Synaptogyrin localizes predominantly to SVs at nerve terminals, as well as to microvesicles in endocrine cell types outside the nervous system^17^. Cell studies have further shown that synaptogyrin regulates Ca^2+^-dependent exocytosis^41^. In mice with a synaptogyrin-synaptophysin double knockout, short-term and long-term synaptic plasticity were reduced^16^. In vivo evidence for a direct role of synaptogyrin in the formation of small vesicles was obtained when a synaptogyrin null mutant was studied in *Drosophila*^13^. The *Drosophila* genome encodes a single synaptogyrin isoform and lacks a synaptophysin homolog, which minimizes compensatory effects associated with synaptogyrin knockouts that might occur in other models systems. The flies that lacked synaptogyrin had SVs with increased diameters^13^. In addition, the flies lacking synaptogyrin showed changes in transmission, that is the study connected synaptogyrin with the diameter of SVs and neurotransmitter-associated function^13^. Further support for the importance of synaptogyrin and synaptophysin for sculpting of small vesicles was provided by ectopic expression of synaptophysin in non-neuronal cells, which induced the formation of small cytoplasmic vesicles^12^. The synaptogyrin-phosphatidylserine-dependent remodeling of liposomes into small vesicles reported in the current work (Fig. 3) demonstrates, through a bottom-up reconstitution approach, that synaptogyrin alone, that is in the absence of other SV proteins, is able to remodel SV-like membranes and induce the formation of SVs. Changes in membrane morphology already occur at synaptogyrin:lipid ratios of 1:500 (Fig. 3a). In addition, when the membrane contains only PC, PS and cholesterol, the vesicle diameter is decreased from 195 to 124 nm at the synaptogyrin:lipid ratio of 1:500 (Extended Data Fig. 9a). Formation of SV-sized vesicles with a diameter of 40–50 nm required synaptogyrin:lipid ratios of 1:50 (Fig. 3a). The data suggest that the high density of proteins in SVs^6^, for example through steric exclusion and the formation of synaptogyrin clusters, might contribute to the formation of 40- to 50-nm-sized SVs.

**Fig. 6:**
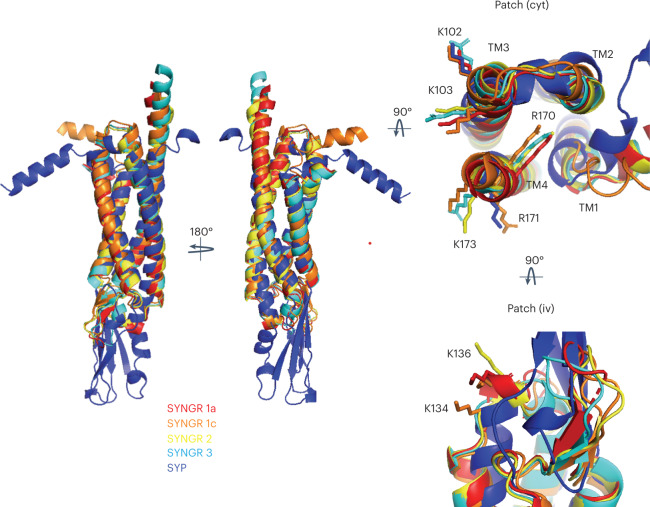
The TM structure of tetraspan vesicle membrane proteins is conserved. Comparison of the structures of different isoforms of synaptogyrin, as well as the structure of synaptophysin (SYP), as predicted by AlphaFold2. When available, the AlphaFold2-predicted structures were downloaded from the AlphaFold Protein Structure Database (www.alphafold.ebi.ac.uk)^45,46^; otherwise, they were predicted using the AlphaFold2 notebook (https://colab.research.google.com/github/sokrypton/ColabFold/blob/main/AlphaFold2.ipynb). Views from the cytoplasmic (cyt) and the intravesicular (iv) membrane side are displayed to the right. Side chains are shown and labeled for selected lysine and arginine residues of synaptogyrin 1b. N- and C-terminal tails were removed for better visualization.

The high sequence conservation between different synaptogyrins, including the two lysine-arginine clusters (Fig. 6), suggests that other synaptogyrins might also be able to remodel the SV membrane in a PS-dependent manner. The related tetraspan vesicle membrane protein synaptophysin, however, lacks some of the lysine and arginine residues (Fig. 6). Future studies will thus have to show whether synaptophysin is able to remodel the membrane of SVs and elucidate the influence of different SV lipids, including cholesterol^8^, that is whether the membrane remodeling activity of synaptogyrins, synaptoporins and synaptophysin might be interchangeable. Such an interchangeability between different tetraspan vesicle membrane proteins in shaping the SV membrane could be responsible for the mild functional deficits observed in synaptogyrin-synaptophysin knockout studies^16,18,41^, and could at the same time ensure that the formation of uniformly sized SVs in the mammalian brain is highly robust. Because the SV membrane contains a high concentration of TM proteins^6^, the membrane-remodeling activity of synaptogyrin, and potentially other tetraspan vesicle membrane proteins, might be amplified by homo- or heterophilic protein interactions or through indirect protein interactions via the bilayer^6,42^.

We determined the high-resolution structure of isoform b of human synaptogyrin 1, a protein from the family of tetraspan vesicle membrane proteins. We further show that binding of PS changes the TM structure of synaptogyrin and is critical for the ability of synaptogyrin to remodel liposomes into small vesicles. Because synaptogyrin has been linked to several diseases of the brain^19–24,43,44^, the findings from our study are likely relevant for both normal and perturbed brain activity.

## Methods

### Molecular cloning, protein expression, and purification

The construct of synaptogyrin 1b (Uniprot: O43759-2) was cloned into the pET16b vector with an N-terminal histidine tag. The synaptogyrin mutants were engineered using site-directed mutagenesis (Thermo Fisher Scientific) and confirmed by DNA sequencing (Supplementary Table 3). Expression was performed in the *Escherichia coli* strain Lemo21 (DE3) with 250 µM l-rhamnose, following previously established procedures^27^. Cells were grown in Luria Bertini or minimal medium containing ^15^NH_4_Cl and/or [^13^C_6_]glucose as the sole nitrogen or carbon source in H_2_O or D_2_O, respectively. Single or double amino acid selective labeling was obtained using [^15^N]Ala, [^15^N]Leu, [^15^N]Cys, [^15^N]Lys, [^15^N]Phe and [^15^N]Tyr as precursors, and the other amino acids were unlabeled in the minimal medium to suppress scrambling. Protein methyl-labeled on Ile^δ1^, Leu^proS^ and Val^proS^ was expressed using ^13^CH_3_-methyl specifically labeled precursors (NMR-Bio) in fully deuterated medium. The precursor of 2-(D_3_) methyl, 2,4-(^13^C_2_) acetolactate was added 1 h prior to the deuterated M9 medium. The 2-ketobutyric acid 4-(^13^C), 3,3 (D_2_) sodium salt was added 20 min before induction by addition of IPTG.

For synaptogyrin production, the temperature was reduced to 20 °C at an optical density at 600 nm (OD_600_) of 0.8–1.0 after seeding the preculture. Cells were induced with 0.2 mM isopropyl β-d-1-thiogalactopyranoside (IPTG) after 1 h and were further incubated at 20 °C for 16 h before collection. The cell pellet was resuspended in 50 ml lysis buffer (20 mM NaPi, pH 7.5, 300 mM NaCl, 1 mM DTT, 1 mg ml^–1^ lysozyme, 0.01 mg ml^–1^ DNaseI, 1 mM phenylmethylsulfonyl fluoride, and one Complete Protease Inhibitor Cocktail-EDTA free (Roche Applied Science)) and homogenized by stirring for 50 min at 4 °C. The suspension was lysed with an Emulsiflex (Avastin) and centrifuged (10,000*g*, 10 min, 4 °C) for removal of cell debris. The membrane fraction was collected from the supernatant by ultracentrifugation (Beckman Ti45 rotor) at 100,000*g* at 4 °C for 50 min. The membranes were resuspended and solubilized by solubilization buffer (20 mM NaPi, pH 7.5, 300 mM NaCl, 1 mM DTT, and one Complete Protease Inhibitor Cocktail-EDTA free, 1 % dodecyl-ß-d-maltoside (DDM)) at 4 °C for 1 h. The solubilized membrane fraction was loaded onto a histidine-trap open column, and the imidazole concentration was increased stepwise to 400 mM. When required, the DDM detergent was exchanged for different detergents, such as 0.005% 2,2-didecylpropane-1,3-bis-β-d-maltopyranoside (NG310) or 0.1% n-undecyl β-maltoside (UDM) in this step. The protein was eluted with 400 mM imidazole, followed by removal of the histidine-tag with TEV protease during dialysis (20 mM sodium phosphate, pH 7.5, 300 mM NaCl, 1 mM DTT, 5% glycerol, 0.03% DDM (0.1% UDM or 0.005% NG310)). After cleavage, the TEV and the histidine tag were removed by loading onto a second histidine-trap column. The pure protein was further purified by gel filtration (HiLoad Superdex 200 column) in 20 mM HEPES, pH 7.5, 150 mM NaCl, 1 mM DTT, 5% glycerol, 0.03% DDM (or 0.1% UDM or 0.005% NG310). The purified protein was verified by SDS polyacrylamide gel electrophoresis. The protein sample buffer was dialyzed against NMR buffer (20 mM sodium phosphate, pH 8.0, 150 mM NaCl, 1 mM TCEP, 0.1 % UDM) for 18 h at 4 °C.

### Circular dichroism

Far-ultraviolet CD measurements were performed at 40 °C using 10 μM synaptogyrin in 20 mM sodium phosphate, pH 7.5, 1 mM DTT, 0.1% UDM on a Chirascan spectrometer (Applied Photophysics). The path length of the cuvette was 0.2 mm. CD spectra were recorded from 190 to 280 nm with an integration time of 0.5 s. The measurement was repeated three times. The final spectrum was obtained by baseline subtraction using the buffer-only measurement.

### Liposome morphology

DLS measurements were performed at 25 °C using a DynaPro NanoStar instrument with a detector positioned at a 90° angle concerning the incident light. The monochromatic laser with a wavelength of 662 nm (auto-attenuation turned on) irradiated the liposome sample in disposable COC cuvettes. Liposome samples (0.4 mM lipid) containing increasing synaptogyrin concentrations were incubated for 18 h at 25 °C in 20 mM HEPES, pH 7.5, and 1 mM TCEP prior to the measurement. DLS data were acquired with 20 cycles and 5 s of acquisition time for three repeated measurements and were analyzed with DYNAMICS v7.10.0.23. Error bars represent the s.d. of three measurements. Microsoft Excel v16.43 was used to fit data, as well as for statistical analysis.

In parallel, samples from the same preparation were adsorbed onto 400-mesh carbon-coated copper grids, and the buffer was removed using filter paper. Samples were then stained by adding 1% uranyl acetate solution, which was dried with a filter paper. The grids were imaged using a FEI Tecnai Spirit electron microscope with a TVIPS F416 4K camera.

### NMR spectroscopy

NMR spectra were recorded at 40 °C on Bruker 600, 700, 800, 900, and 950 MHz Bruker NMR spectrometers using cryogenic probes. ^1^H spin-spin echo experiments with echo times from 0.1 to 8.1 ms were recorded on 0.1–0.3 mM synaptogyrin in 20 mM sodium phosphate, pH 8.0, 150 mM NaCl, 1 mM TCEP, 0.1% UDM (or 0.005% NG310 or 0.03% DDM) at 40 °C. Relaxation times (*T*_2_) were calculated using peak intensities of NMR signals between 9.5 ppm and 8.5 ppm.

The backbone resonances were assigned by TROSY-based 3D triple resonance through bond scalar correlation HNCO, HN(CA)CO, HNCA, HN(CO)CA, and HN(CA)CB experiments^47–49^ using ^2^H-^13^C-^15^N-labeled synaptogyrin (0.8 mM synaptogyrin in 20 mM sodium phosphate, pH 8.0, 150 mM NaCl, 1 mM TCEP, 0.1 % UDM). Backbone resonance assignment was further supported by a 3D ^15^N-edited NOESY-HSQC (NOE mixing time of 150 ms) experiment recorded on 0.8 mM ^2^H-^15^N-labeled synaptogyrin in 20 mM sodium phosphate, pH 8.0, 150 mM NaCl, 1 mM TCEP, 0.1% UDM, as well as ^1^H-^15^N TROSY-HSQC experiments recorded on [^15^N]Ala/[^15^N]Leu-, [^15^N]Leu/[^15^N]Tyr]-, [^15^N]Cys/[^15^N]Phe-, [^15^N]Thr-, and [^15^N]Lys-labeled synaptogyrin (0.8–1.0 mM). Side chain assignments were determined using a 3D (H)CCH-TOCSY (mixing time 10 ms), two 3D ^13^C-edited NOESY-HSQC (80 ms and 300 ms mixing time; 1.0 mM ^13^C-^15^N-labeled synaptogyrin in 20 mM sodium phosphate, pH 8.0, 150 mM NaCl, 1 mM TCEP, 0.03 % d25-DDM, 100% D_2_O), and two 3D ^15^N-edited NOESY-HSQC (80 ms and 300 ms mixing time; 0.9 mM ^15^N-labeled synaptogyrin in 20 mM sodium phosphate, pH 8.0, 150 mM NaCl, 1 mM TCEP, 0.03 % d25-DDM, 10 % D_2_O) experiments. For [U-^2^H-^15^N; I^δ1^ /LV^proS 13^CH_3_]-labeled synaptogyrin, 2D ^1^H-^13^C HSQC and ^13^C-edited NOESY-HSQC (200 ms mixing time) experiments were recorded and used for the assignment of the methyl groups of the Ile, Leu and Val residues. Long-range NOE restraints between HN backbone resonances and side chain methyl protons were extracted from 3D ^15^N-resolved NOESY-HSQC experiments. recorded for 0.4 mM [U-^2^H-^15^N; I^δ1^ /LV^proS 13^CH_3_]-labeled synaptogyrin in 20 mM sodium phosphate, pH 8.0, 150 mM NaCl, 1 mM TCEP, 0.03% d25-DDM, 10% D_2_O.

Residual ^1^D_NH_ dipolar couplings were obtained by taking the difference in the J splitting values measured in oriented (7% acrylamide gel alignment medium) and isotropic sample conditions using interleaved 2D ^1^H-^15^N TROSY-HSQC/2D ^1^H-^15^N HSQC spectra recorded on 0.8 mM ^2^H-^15^N-labeled synaptogyrin in 20 mM sodium phosphate, pH 8.0, 300 mM NaCl, 1 mM TCEP, 0.1% UDM. NMR spectra were processed with Topspin 3.6.1 (Bruker) and analyzed using the software CcpNmr (Analysis 2.4.2)^50^. Residue-specific secondary structure scores and random coil indices (RCI S^2^) were determined from the assigned NMR chemical shifts of synaptogyrin using TALOS+ (ref. ^51^). The RDC values were analyzed using the PALES program^52^.

For the titration experiments of wild-type and mutant synaptogyrin with DMPS, 0.2 mM ^2^H-^15^N-labeled synaptogyrin was solubilized in isotropic bicelles without or with 20 % DMPS ((DMPC:DMPS = 8:2)/DHPC, *q* = 0.3) corresponding to a protein:DMPS ratio of 1:40. The buffer was 20 mM sodium phosphate, pH 8.0, 150 mM NaCl, 1 mM TCEP. ^1^H and ^15^N normalized weighted average chemical shift perturbations were calculated using Δδ*NH* = [((Δ*H*)^2^ + (Δ*N*)^2^ / 5) / 2]^1/2^, where $\Delta H$ and $\Delta N$ correspond to the ^1^H and ^15^N chemical shift differences between two states (for example, without and with DMPS).

To measure *T*_2_, we used a 90°–180° pulse sequence. Fitting the intensity decay curve to *I*_0_ (–t / T_2_) with *I*_0_ being the starting intensity gives the characteristic time constant *T*_2_. The correlation time τ_c_ was subsequently calculated according to:

$$\frac{1}{T2}=\frac{K}{2}\left[3{\tau }_{c}+\frac{5{\tau }_{c}}{1+\,{\omega }_{0}^{2}{\tau }_{c}}+\frac{{\tau }_{c}}{1+4{\omega }_{0}^{2}{\tau }_{c}}\right]$$

$$K=\frac{3{\mu }_{0}^{2}}{160{\pi }^{2}}\frac{{\hslash }^{2}{\gamma }^{4}}{{r}^{6}}.$$

The relaxation times are approximately associated with the correlation time by the equations according to Bloembergen–Purcell–Pound (BPP) theory, where *ω*_0_ is the rotational frequency of the signal, *µ*_0_ is the magnetic permeability of free space (4π × 10^−7^ H m^−1^), *ħ* is the reduced Planck constant 1.054571726 × 10^−34^ J s^–1^, *γ* is the gyromagnetic ratio of the nucleus (for ^1^H, it is 2.67513 × 108 rad s^−1^ T^−1^) and *r* is the distance between magnetically active spin-½ nuclei. The molecular weight of synaptogyrin in different micelle conditions was then calculated according to:

$${\mathrm{Molecular}}\; {\mathrm{weight}}=\frac{{\tau }_{c}{\mathrm{RT}}}{\eta (v+h)}$$

*η* is viscosity, *v* is the partial specific volume, *h* is the degree of hydration, R is the ideal gas constant and *T* is temperature.

### Paramagnetic relaxation enhancement

For the spin labeling on Cys124 of synaptogyrin with MTSL (S-(1-oxyl-2,2,5,5-tetramethyl-2,5-dihydro-1H-pyrrol-3-yl)methyl methanesulfonothioate), the three other native cysteines in synaptogyrin were mutated to either serine or alanine (C59A C68A C82S). To remove residual reducing agent, synaptogyrin was passed through a desalting column (HiPrep 26/10 Desalting; GE Healthcare). The protein was collected into a solution containing tenfold excess of MTSL and incubated at 4 °C for 18 h. Using the desalting column, we removed the excess MTSL, then exchanged the protein into NMR buffer containing 20 mM NaPi, pH 8.0, 150 mM NaCl, and 0.1% UDM. To determine the paramagnetic relaxation enhanced-broadening of the NMR signals, a ^1^H-^15^N TROSY-HSQC experiment was recorded for 1.0 mM ^15^N-labeled mutant synaptogyrin (C59A C68A C82S) in 20 mM sodium phosphate, pH 8.0, 150 mM NaCl, 1 mM TCEP, 0.1 % UDM.

To probe the hydrophobic burial and solvent exposure of synaptogyrin residues, we used the detergent-soluble 16-doxylstearic acid (16-DSA) and the water soluble [5,8-bis-(carboxymethyl)-11-[2-(methylamino)-2-oxoethyl]-3-oxo-2,5,8,11-tetraazatridecan-13-oato(3-)]-gadolinium (gadodiamide), respectively. The paramagnetic reagents were stepwise added to 0.8 mM U-[^2^H-^15^N]-synaptogyrin solubilized in UDM micelles. ^1^H-^15^N TROSY-HSQC spectra were then recorded at 40 °C using Bruker 900 and 800 MHz spectrometers.

### Structure calculation

The recorded backbone chemical shifts (HN, N, C, Cα and Cβ) of residues 16–176 of synaptogyrin were used in RASREC CS-Rosetta to select 200 fragments of 3 and 9 residues in length. The N- and C-terminal tails of synaptogyrin were not included into the structure determination process, because they were predicted to be disordered by TALOS+ (ref. ^51^) on the basis of the experimental NMR chemical shifts. In addition, the loop regions formed by residues 47–65, 91–98 and 132–143, which had a TALOS+-predicted order parameter of less than 0.7, were removed from the Rosetta score computation, but were part of the structure calculation. NOE restraints from 3D ^15^N-edited NOESY-HSQC experiments were manually assigned. In the later stages of the structure calculation, NOE restraints determined automatically by CYANA were also included. The NOE-based distances (*d*_0_) were restrained with a ROSETTA flat-bottom potential with fixed lower bound (lb) = 1.5, upper bound (ub) = *d*_0_ + 0.15 Å, and inverse curvature (*c*) = 0.3 Å. In addition, 68 long-range PRE-based distances were restrained between the C_β_ atom of the MTSL-labeled residue (Cys124) and the corresponding backbone amide protons with error bounds of ±4 Å in order to account for the size and flexibility of the Cys-MTSL^53^. NOE, PRE and RDC data maintained weights of 5 and 0.1 for scoring in the low-resolution sampling stage and the all-atom sampling stage, respectively. A weight equal to 5 was used for the NOE, PRE and RDC data for the centroid and all-atom structures to select a pool of iterated structures. The standard Rosetta all-atom energy function was used with the experimental weight set mpframework_smooth^54^. The energy function was adjusted for TM proteins to model the hydrophobic TM region^55^. TM regions were determined from the amino acid sequence using the OCTOPUS method^56^. Five thousand structures were calculated, and the ten structures with the lowest Rosetta all-atom energy score were selected for further analysis. Structure figures were prepared using the PyMOL Molecular Graphics System (Version 1.8.2.1).

### Liposome assays

Liposomes were formed from L-α-phosphatidylcholine (PC), L-α-phosphatidylethanolamine (PE), L-α-phosphatidylserine (PS), L-α-phosphatidylinositol (PI), and cholesterol (Avanti Polar Lipids) in selected wt/wt ratios. After addition of each phospholipid to the glass tube, the phospholipid mixture was evaporated off chloroform-methanol (1:1) under a low stream of nitrogen. The organic solvent was lyophilized to obtain a thoroughly dried lipid film. The dry lipid film was hydrated with 1 mL of liposome buffer (20 mM NaPi HEPES, pH 7.5, 1 mM TCEP). To produce unilamellar liposomes, the rehydrated lipid was sonicated three times for 2 min using an ultrasonic water bath sonicator. Next, the lipid film was extruded through a filter with a 200-nm pore size and thus liposomes of more uniform size were obtained. In order to reconstitute DDM-solubilized synaptogyrin into liposomes, DDM was then removed using Bio-Beads SM-2 Resin (Bio-rad). After DDM removal, the synaptogyrin sample was directly added to the liposomes. The sample was incubated for 18 h at 25 °C in 20 mM HEPES, pH 7.5, 1 mM TCEP. For NMR experiments, the sample was further concentrated using 30-kDa cut-off vivaspin (Satorious). NMR spectroscopy was used to confirm removal of DDM.

### Reporting summary

Further information on research design is available in the Nature Portfolio Reporting Summary linked to this article.

## Online content

Any methods, additional references, Nature Portfolio reporting summaries, source data, extended data, supplementary information, acknowledgements, peer review information; details of author contributions and competing interests; and statements of data and code availability are available at 10.1038/s41594-023-01004-9.

## Supplementary information

Supplementary Information Supplementary Tables 1–3 and Figures 1 and 2.

Reporting Summary

## Data Availability

The structure of synaptogyrin has been deposited in the Protein Data Bank (PDB) under the accession number 8A6M; the corresponding NMR restraints were deposited in the Biological Magnetic Resonance Bank (BMRB) under the accession number 34738. The AlphaFold2-predicted structures of synaptogyrin 1a (ID O43759), synaptogyrin 2 (ID K7ENG9), synaptogyrin 3 (ID O43761) and synaptophysin (ID P08247) were downloaded from the AlphaFold Protein Structure Database (www.alphafold.ebi.ac.uk). Source data for Figures 1c, 3b, 4b and 5a and Extended Data Figs. 2, 5b, 6c, 9a and 10b are available for this paper.

## Source data

Source Data Fig. 1 Endnote file with data.
Source Data Fig. 3 Endnote file with data.
Source Data Fig. 4 Endnote file with data.
Source Data Fig. 5 Endnote file with data.
Source Data Extended Data Fig. 2 Endnote files with data.
Source Data Extended Data Fig. 5 Endnote file with data.
Source Data Extended Data Fig. 6 Endnote file with data.
Source Data Extended Data Fig. 9 Endnote file with data.
Source Data Extended Data Fig. 10 Endnote file with data.
