# Supplementary material for: Phosphatidylserine-dependent structure of synaptogyrin remodels the synaptic vesicle membrane
Source: Nat Struct Mol Biol. 2023 May 22;30(7):926–34. doi: 10.1038/s41594-023-01004-9 (PMC10352133; doi:10.1038/s41594-023-01004-9)
Supplement: Supplementary file 1 — Supplementary Tables 1–3 and Figures 1 and 2. [file 41594_2023_1004_MOESM1_ESM.pdf]

nature structural & molecular biology

Article

https://doi.org/10.1038/s41594-023-01004-9

Phosphatidylserine-dependent structure of synaptogyrin remodels the synaptic vesicle membrane

In the format provided by the authors and unedited

Supporting Information

Supporting Tables 1-3
Supporting Figures 1-2

Supporting Information Table 1. Overview providing information about the recorded NMR experiments, samples and spectrometers.

	Sample labeling	NMR experiment	Spectrometer (MHz)	TD	Measurement time (day)
**Backbone/side-chain Assignment + NOE Restraints**	^{15}N	3D ^1H-^{15}N trNOESY	900	2048 X 64 X 128	5
	^{13}C, ^{15}N	3D ^1H-^{13}C NOESY	900	2048 X 64 X 128	7
		3D aromatic NOESY	950	2048 X 64 X 128	7
		3D HCCH TOCSY	950	2048 X 64 X 128	5
		3D HAHN	950	2048 X 40 X 128	5
		2D ^1H-^{13}C HSQC	950	2048 X 256	5
	^2H, ^{15}N	2D ^1H-^{15}N trHSQC	950	2048 X 256	0.5
	^2H, ^{15}N	3D ^1H-^{15}N trNOESY	900	2048 X 54 X 130	5
	^2H, ^{13}C, ^{15}N	3D NUS-trHNCO	950	2048 X 64 X 128	3
		3D NUS-trHN(CA)CO	950	2048 X 64 X 128	4
		3D NUS-trHNCA	950	2048 X 64 X 128	3
		3D NUS-trHN(CO)CA	950	2048 X 48 X 160	4
		3D NUS-trHNCB	950	2048 X 64 X 128	4
	^2H, ^{15}N; Iδ1 /LVproS $^{13}CH_3$	2D ^1H-^{13}C HSQC	950	2048 X 256	0.5
		3D ^1H-^{13}C NOESY	950	2048 X 256	7
	^{15}N-Ala/^{15}N-Leu	2D ^1H-^{15}N trHSQC	900	2048 X 256	0.5
	^{15}N-Leu/^{15}N-Tyr	2D ^1H-^{15}N trHSQC	900	2048 X 256	0.5
	^{15}N-Cys/^{15}N-Phe	2D ^1H-^{15}N trHSQC	800	2048 X 256	0.5
	^{15}N-Thr	2D ^1H-^{15}N trHSQC	950	2048 X 256	0.5
	^{15}N-Lys	2D ^1H-^{15}N trHSQC	950	2048 X 256	0.5
**Titration**	^2H, ^{15}N	2D ^1H-^{15}N trHSQC	800	2048 X 256	1
**RDC**	^2H, ^{15}N	2D ^1H-^{15}N HSQC	800	2048 X 256	2
**PRE**	^2H, ^{15}N	2D ^1H-^{15}N trHSQC	800/700	2048 X 256	1

Supporting Information Table 2. Long-range NOEs in the transmembrane region with the corresponding distances (in Å) in the lowest Rosetta energy-structure of synaptogyrin.

TM	Residue		Residue		Distance
**TM1**	16	H	174	HB3	4.15
	18	H	175	QD2	3.22
	18	QE	94	H	4.83
	19	H	175	QD2	3.35
	21	H	172	H	5.5
	21	QG1	94	H	4.25
	25	H	167	QD2	3.41
	28	H	164	QG2	3.07
	28	QD1	164	H	4.05
	29	HG2	86	H	6.21
	29	H	164	QG2	5.28
	30	H	86	QD1	3.18
	30	H	86	HA	7.11
	31	QG2	160	H	6.1
	32	H	160	HA	6.25
	32	H	161	HB3	8.23
	33	H	82	HB3	4.44
	35	QE	157	H	4.6
	36	H	79	QD	5.35
	38	H	153	HB3	6.11
	38	H	153	QE	6.03
	39	H	153	QE	7.26
	39	QG1	154	H	5.49
	40	QD	73	H	8
	42	HB2	149	H	5.92
	43	H	153	HB2	4.15
	43	H	153	QE	4.13
	43	H	146	QB	4.25
	43	H	76	QG1	4.44
	44	QG2	71	H	8.17
	44	QG2	73	H	6.88
	45	H	143	HA1	4.31
**TM2**	66	H	129	HG3	6.48
	66	H	126	HA	9.44
	66	HB3	126	H	9.26
	67	H	126	HG	6.06
	70	QD	122	H	5.4
	71	H	126	QD1	3.78
	74	H	118	QD1	6.18
	74	H	76	H	4.51
	74	QG1	119	H	5.17
	75	H	119	HZ3	4.13
	76	QG1	44	H	3.7
	76	H	40	QD	4.7
	77	H	115	HZ2	5.13
	78	H	115	HE3	6.17
	80	H	115	HZ3	6.17
	81	H	111	HB	9.4
	81	H	111	HA	9.61
	89	H	26	QG2	6.67
**TM3**	102	H	175	QD1	3.56
	102	H	175	HB2	5.33
	102	HG2	176	H	5.01
	102	HB2	176	H	6.08
	104	H	88	QD1	5.78
	105	H	172	QD	4.68
	105	H	88	QD1	5.21
	109	H	168	QB	3.97
	113	H	162	HB2	8.3
	113	H	165	QB	3.88
	115	H	161	HE3	4.39
	116	H	161	HD1	5.56
	120	H	158	QD1	4.43
	122	H	74	QG1	5.26
	126	H	67	QB	5.42
	127	H	151	QD1	4.11
**TM4**	146	QB	45	H	4.22
	146	QB	46	H	3.94
	146	H	46	HB2	4.13
	150	H	39	HA	4.23
	150	H	42	HB2	4.41
	151	H	126	HB2	5.15
	152	H	123	QE	5.44
	153	H	38	QG1	4.34
	154	H	123	HB2	5.49
	155	H	123	QD	3.32
	158	H	119	HB2	3.58
	161	HB2	115	H	4.54
	162	H	116	HA	4.28
	163	H	28	QD1	5.28
	165	QB	112	H	5.28
	165	H	112	HB2	2.95
	168	QB	110	H	6.32
	172	H	105	QG1	3.22
	175	QD1	103	H	5.24

Supporting Information Table 3. Primer sequence for synaptogyrin mutants

Mutant	Mutated site	Forward primer	Reverse primer
SYNGR1b C67A, C82S, C124A	C67A	AACCCCAACGCCGCCAGCTATGGCGTG	CACGCCATAGCTGGCGGCGTTGGGGTT
	C82S	GTGCTCGCCTTCCTCACCAGCCTGCTGTACCTGGCCCTG	CAGGGCCAGGTACAGCAGGCTGGTGAGGAAGGCGAGCAC
	C124A	TGGTTCGTGGGATTCGCCTACCTGGCCAAC	GTTGGCCAGGTAGGCGAATCCCACGAACCA
SYNGR1b K102Q	K102Q	AGCCTCAAGGACCGCCAGAAAGCCGTCCTG	CAGGACGGCTTTCTGGCGGTCCTTGACGCT
*SYNGR1b K102, 103Q	K103Q	AAGGACCGCCAGCAGGCCGTCCTG	CAGGACGGCCTGCTGGCGGTCCTT
SYNGR1b K134Q	K134Q	TGGCAGGTCTCCCAGCCCAAGGACAAC	GTTGTCCTTGGGCTGGGAGACCTGCCA
*SYNGR1b K134, 136Q	K136Q	CAGGTCTCCCAGCCCCAGGACAACCCA	TGGGTTGTCCTGGGGCTGGGAGACCTG

* The double mutated SYNGR1b_K102,103Q and K134, 136Q used pDNA of SYNGR1b K102Q, K134Q, respectively.

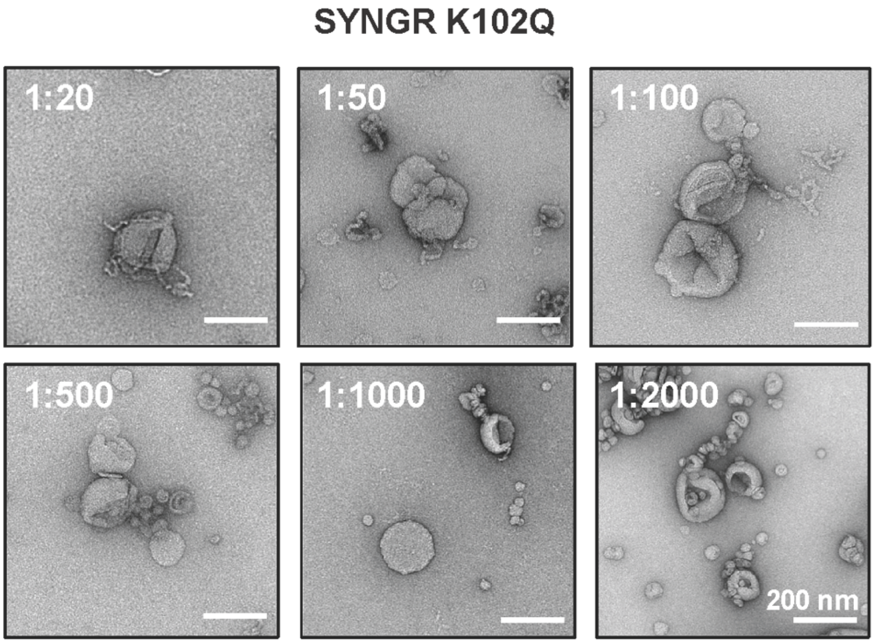

Supporting Information Fig. 1 | Negative-stain electron micrographs of liposomes/vesicles (PC:PE:PS:PI:cholesterol = 40:32:12:5:10) with decreasing concentrations of mutant K102Q synaptogyrin; scale bar, 200 nm. Protein:lipid molar ratios are indicated.

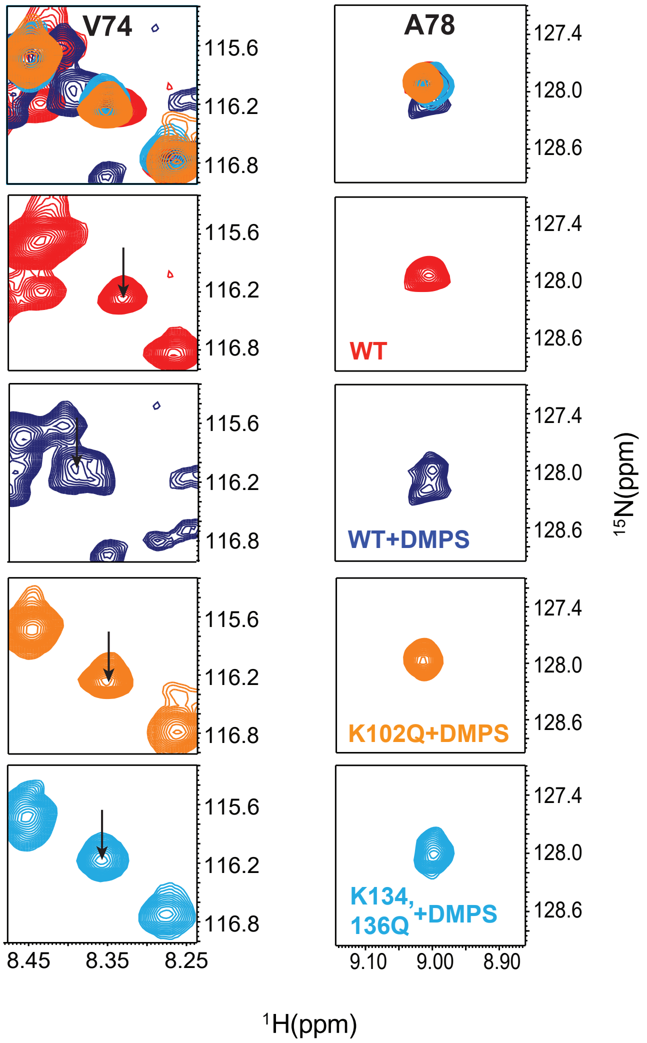

Supporting Information Fig. 2 | Selected regions from the ^{1}H-^{15}N TROSY-HSQC spectra of $^{15}N/^{2}H$-labeled wild-type synaptogyrin in isotropic bicelles (DMPC/DHPC, q = 0.3) without (red) or with 20% DMPS (blue), as well as the two $^{15}N/^{2}H$-labeled synaptogyrin mutants in isotropic bicelles (DMPC/DHPC, q = 0.3) in the presence of 20% DMPS (K102Q synaptogyrin, orange; K134Q/K136Q synaptogyrin, cyan). The top panels show the superpositions, while the panels below display the individual spectral regions.
