# Supplementary material for: Phosphatidylserine-dependent structure of synaptogyrin remodels the synaptic vesicle membrane
Source: Nat Struct Mol Biol. 2023 May 22;30(7):926–34. doi: 10.1038/s41594-023-01004-9 (PMC10352133; doi:10.1038/s41594-023-01004-9)
Supplement: Supplementary file 2 — Reporting Summary [file 41594_2023_1004_MOESM2_ESM.pdf]

nature research

Corresponding author(s): Markus Zweckstetter

Last updated by author(s): Mar 13, 2023

Reporting Summary

Nature Research wishes to improve the reproducibility of the work that we publish. This form provides structure for consistency and transparency in reporting. For further information on Nature Research policies, see our Editorial Policies and the Editorial Policy Checklist.

Statistics

For all statistical analyses, confirm that the following items are present in the figure legend, table legend, main text, or Methods section.

n/a	Confirmed	
☐	☒	The exact sample size (*n*) for each experimental group/condition, given as a discrete number and unit of measurement
☐	☒	A statement on whether measurements were taken from distinct samples or whether the same sample was measured repeatedly
☐	☒	The statistical test(s) used AND whether they are one- or two-sided<br>*Only common tests should be described solely by name; describe more complex techniques in the Methods section.*
☒	☐	A description of all covariates tested
☒	☐	A description of any assumptions or corrections, such as tests of normality and adjustment for multiple comparisons
☐	☒	A full description of the statistical parameters including central tendency (e.g. means) or other basic estimates (e.g. regression coefficient) AND variation (e.g. standard deviation) or associated estimates of uncertainty (e.g. confidence intervals)
☒	☐	For null hypothesis testing, the test statistic (e.g. *F*, *t*, *r*) with confidence intervals, effect sizes, degrees of freedom and *P* value noted<br>*Give P values as exact values whenever suitable.*
☒	☐	For Bayesian analysis, information on the choice of priors and Markov chain Monte Carlo settings
☒	☐	For hierarchical and complex designs, identification of the appropriate level for tests and full reporting of outcomes
☒	☐	Estimates of effect sizes (e.g. Cohen's *d*, Pearson's *r*), indicating how they were calculated

Our web collection on statistics for biologists contains articles on many of the points above.

Software and code

Policy information about availability of computer code

Data collection	NMR data were acquired using Topspin 3.5pl7, 4.0.7, and 4.0.8; DLS data were acquired with DYNAMICS v7.10.0.23; CD was measured using a Chirascan spectrometer; Electron microscopy experiment were acquired using a FEI Tecnai Spirit electron microscope with TVIPS F416 4K camera.
Data analysis	NMR data were processed with Topspin 3.6.1 and analyzed using CcpNmr (Analysis 2.4.2); Protein structure was calculated using RASREC CS-ROSETTA; Secondary structure was determined using TALOS+; RDC values was analyzed using PALES (10.4.8 version); DLS data were analyzed with DYNAMICS v7.10.0.23; Microsoft Excel v16.43 was used for fitting data as well as for statistical analysis. Structure figures were prepared using the PyMOL Molecular Graphics System (Version 1.8.2.1). When available the AlphaFold2-prediced structures were downloaded from the AlphaFold Protein Structure Database (www.alphafold.ebi.ac.uk), otherwise predicted using the AlphaFold2 notebook (colab.research.google.com/drive/1LVPSOf4L502F21RWBmYJJYYLDlOU2NTL).

For manuscripts utilizing custom algorithms or software that are central to the research but not yet described in published literature, software must be made available to editors and reviewers. We strongly encourage code deposition in a community repository (e.g. GitHub). See the Nature Research guidelines for submitting code & software for further information.

Data

Policy information about availability of data

All manuscripts must include a data availability statement. This statement should provide the following information, where applicable:

- Accession codes, unique identifiers, or web links for publicly available datasets
- A list of figures that have associated raw data
- A description of any restrictions on data availability

The structure of synaptogyrin has been deposited in the Protein Data Bank (PDB) under the accession number 8A6M; the corresponding NMR restraints in the

Biological Magnetic Resonance Bank (BMRB) under the accession number 34738. The AlphaFold2-prediced structures of synaptogyring 1a (ID O43759), synaptogyrin 2 (ID K7ENG9), synaptogyrin 3 (ID O43761) and synaptophysin (ID P08247) were downloaded from the AlphaFold Protein Structure Database (www.alphafold.ebi.ac.uk). Extended data including source data are available for this paper at www.nature.com/nature. Other data that support the findings of this study are available from the corresponding author upon request.

Field-specific reporting

Please select the one below that is the best fit for your research. If you are not sure, read the appropriate sections before making your selection.

☒ Life sciences ☐ Behavioural & social sciences ☐ Ecological, evolutionary & environmental sciences

For a reference copy of the document with all sections, see nature.com/documents/nr-reporting-summary-flat.pdf

Life sciences study design

All studies must disclose on these points even when the disclosure is negative.

Sample size	No calculations were performed to predetermine sample size. NMR data reported for backbone/side-chain assignment, PRE, RDC, NOE, and chemical shift perturbations come mostly from one NMR spectrum in the specified condition.
Data exclusions	No data were excluded while reporting this study.
Replication	DLS data were repeated three times. All attempts of replication were successful. Error bars represent std.
Randomization	Randomization in terms of randomized sampling was not relevant, because we did not select samples meant to represent larger populations.
Blinding	Blinding was not relevant to this study, because decision-making has no impact on the experiment and there is no risk of bias.

Reporting for specific materials, systems and methods

We require information from authors about some types of materials, experimental systems and methods used in many studies. Here, indicate whether each material, system or method listed is relevant to your study. If you are not sure if a list item applies to your research, read the appropriate section before selecting a response.

Materials & experimental systems

n/a	Involved in the study	
☒	☐	Antibodies
☒	☐	Eukaryotic cell lines
☒	☐	Palaeontology and archaeology
☒	☐	Animals and other organisms
☒	☐	Human research participants
☒	☐	Clinical data
☒	☐	Dual use research of concern

Methods

n/a	Involved in the study	
☒	☐	ChIP-seq
☒	☐	Flow cytometry
☒	☐	MRI-based neuroimaging
